# Psychometric evaluation and clustering of the Chinese Community Integration Questionnaire-Revised (CIQ-R) among older adults: a cross-sectional study

**DOI:** 10.3389/fpubh.2026.1753681

**Published:** 2026-02-04

**Authors:** Yuanfang Wang, Xinglan Qin, Lei Xu, Lisha Zheng, Xiaohui Xie, Jia Wang, Wansha Zhou, Lianhong Wang

**Affiliations:** 1 Nursing Department, Affiliated Hospital of Zunyi Medical University, Zunyi, Guizhou, China; 2 Nursing College, Zunyi Medical University, Zunyi, Guizhou, China

**Keywords:** CIQ-R, clustering, community integration, older adults, psychometrics

## Abstract

**Background:**

Reliable instruments for assessing community integration among older adults are limited in China. The Community Integration Questionnaire–Revised (CIQ-R) was translated into Chinese and culturally adapted for use in this population. Its psychometric properties were evaluated, and its practical utility for classifying levels of community integration was examined.

**Methods:**

Following forward–backward translation and expert review, the Chinese Community Integration Questionnaire (CIQ)-R was administered to 400 community-dwelling older adults in Zunyi (April–July 2024). Item analysis, exploratory factor analysis (EFA), and confirmatory factor analysis (CFA) assessed scale structure. Internal consistency was evaluated with Cronbach’s *α*. *K*-means clustering identified integration profiles and empirical cut-offs; cluster validity and between-group differences were examined with nonparametric tests and effect sizes.

**Results:**

The adapted CIQ-R consists of four subscales (Family Integration, Social Integration, Productivity, Electronic Social Networking) and 17 items, following the removal of one poorly performing item (item 2.4). Cronbach’s *α* for the full scale was 0.816. EFA (KMO = 0.808; Bartlett’s *p* < 0.001) yielded a four-factor structure accounting for 61.25% of the variance, with eigenvalues >1; CFA provided support for the proposed model CFA provided support for the proposed model (*χ*^2^ = 268.34, df = 84, *p* < 0.001; *χ*^2^/df = 3.19; CFI = 0.920; TLI = 0.900; RMSEA = 0.074, 90% CI: 0.064–0.084), indicating an acceptable overall model fit. *K*-means clustering identified three distinct and stable profiles (Low, Moderate, and High); empirical thresholds of 13.25 and 21 distinguished the groups (all pairwise comparisons *p* < 0.001).

**Conclusion:**

The Chinese CIQ-R demonstrates acceptable reliability and structural validity and provides a practical tool for identifying heterogeneity in community integration among older adults, with potential utility for population screening and community-based public health planning.

**Clinical trial registration:**

This study was registered with the Chinese Clinical Trial Registry (ChiCTR2300071478).

## 1 Introduction

Population ageing is accelerating worldwide and poses major challenges for health and social systems. China, with the largest older population globally, is experiencing these challenges with particular intensity (1). By the end of 2024, the number of adults aged 60 years and above, a commonly used threshold in China and widely adopted in WHO reports (2), reached 310.31 million, representing 22.0% of the national population (3). Longer life expectancy reflects social progress, yet many older adults face challenges as social roles shrink and daily functioning declines (4).

As people age, they face increased risks of ageism (5) and a shrinking social network (6), leading to increased social isolation. Changes in family structure in China, particularly the rise of “empty-nest” households (7), have contributed to weakening traditional informal support systems. Emotional or psychological mistreatment may further distance older adults from community life, amplifying risks of neglect, depression, and self-harm (8). Loneliness, closely related to but distinct from social isolation, reflects a deeper emotional void (9), while social isolation refers to the lack of social connections. Both are interrelated and negatively impact the physical and mental health of older adults, as well as their social functioning (10). Recent data from China reveals widespread social isolation, a strong sense of loneliness, and high suicide rates among older adults (11, 12), conditions closely linked to higher morbidity and mortality rates (13).

Strengthening social functioning has therefore become a central public health goal. Global initiatives—including the WHO Decade of Healthy Ageing (2) and the Ottawa Charter—encourage societies to create environments that support participation and meaningful roles in later life. Across these efforts runs a common message: community integration is fundamental to healthier ageing trajectories.

Community integration generally refers to individuals’ effective participation in, and fulfillment of, roles within their community. Despite variation in definitions, most frameworks conceptualize community integration as a multidimensional construct. The International Classification of Functioning (ICF) (14) describes participation as “involvement in life situations,” covering family and household integration, community and social participation, and occupational or productive roles. These domains form the core of active ageing (15).

**Figure 1:**
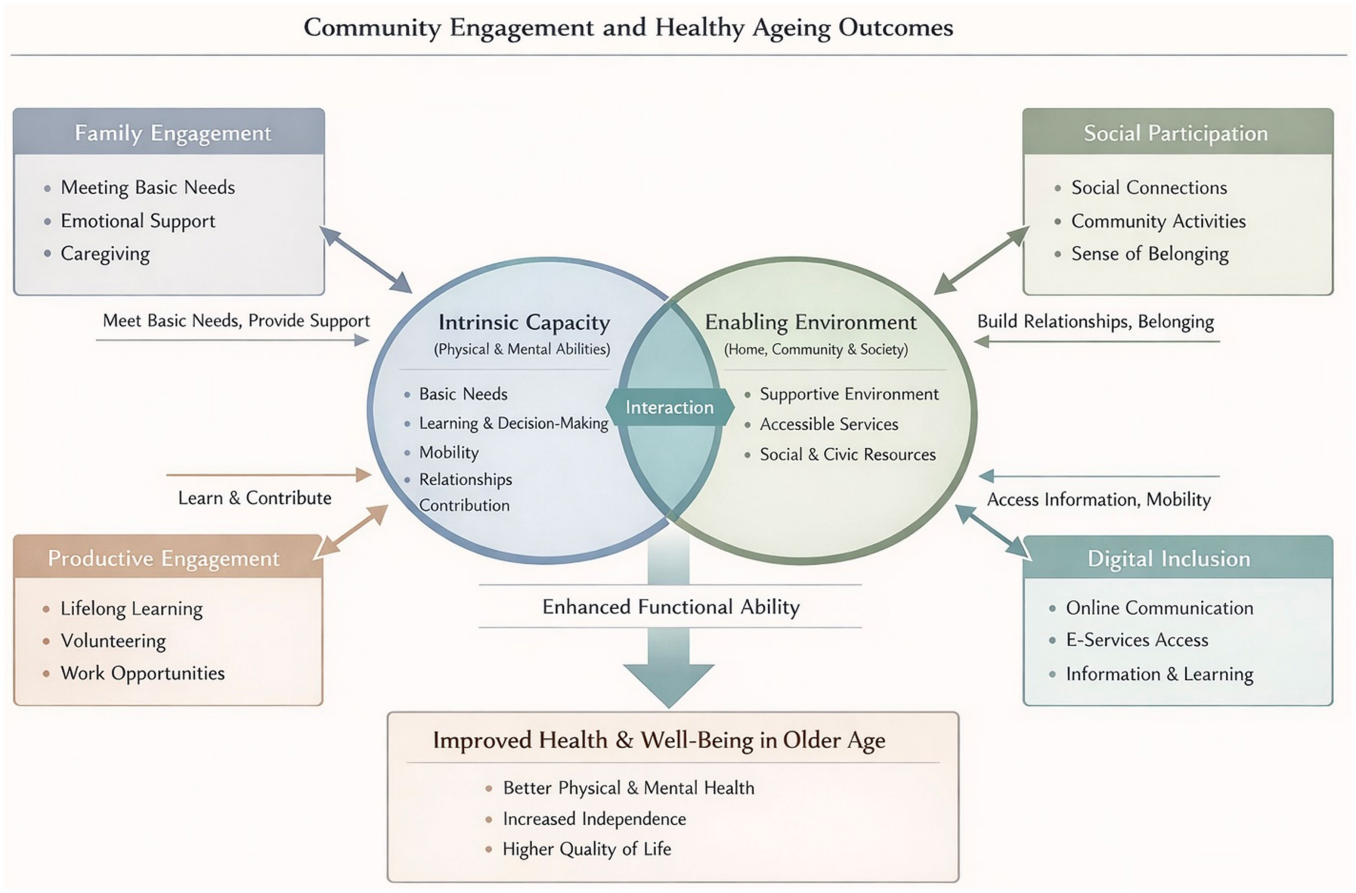
Theoretical framework of community integration and healthy ageing outcomes.

To gain a deeper insight into the ways community integration influences health outcomes (healthy ageing), we draw on the World Health Organization Healthy Ageing framework (16) (Figure 1). The framework centers on a simple but powerful idea: what shapes an older person’s health is not only the presence of disease, but the ability to maintain and build functional capacity. This capacity grows out of the interplay between intrinsic abilities (e.g., mobility and cognition) and the environments that surround daily life. These environments, from family and neighborhood settings to broader social structures, do far more than provide context; they actively shape how intrinsic abilities are expressed, supported, and sometimes compensated.

Within this framework, community integration represents a critical pathway linking environment to functional ability. The degree of connection to family, the depth of social ties, continued engagement in productive roles, and the ability to navigate digital networks together determine how older adults stay involved with the world around them. Higher levels of community integration are associated with stronger social support, more meaningful participation, and improved access to community resources. Accumulating evidence indicates that social participation and a sense of belonging are linked to better mental health, slower cognitive decline, and reduced mortality risk (17). Thus, fostering community integration is not merely a social ideal but a practical mechanism that buffers age-related functional decline and supports healthier ageing trajectories.

Recognizing the pivotal role of community integration, the Chinese government has prioritized increasing community participation among older adults as a key strategy to maintain social functioning (18). Despite policy momentum in China, substantial gaps remain. While existing studies have explored community integration in specific groups—such as individuals with brain injuries, migrants, or people with disabilities (19–21) —understanding of this concept across the broader older adult population in China remains limited. Research within China has largely been confined to the field of social work, focusing on small-scale interventions (22), which limits its generalizability and ability to reflect broader population patterns. A critical barrier is the absence of a reliable, culturally adapted tool for assessing community integration among older Chinese adults. Existing instruments show limited cultural relevance or insufficient psychometric validation.

The Community Integration Questionnaire-Revised (CIQ-R) (23) is an updated form of the original Community Integration Questionnaire (CIQ) (24). The CIQ was developed by Willer et al. (24) for individuals with traumatic brain injury, but it has since been applied to many other populations and has consistently shown strong psychometric performance (25, 26). To address emerging forms of social participation in the digital era and overcome limitations of the original scale, Willer et al. (24) added a three-item Electronic Social Networking subscale, creating the CIQ-R. Versions of the CIQ-R have been adapted for Malay and Italian populations (27, 28), but its suitability for older adults in China has not been tested.

This study aims to: (1) translate and culturally adapt the CIQ-R for use in China; (2) assess its applicability and conceptual alignment for older Chinese adults; (3) evaluate its reliability and validity in this population; (4) apply data-driven cluster analysis to better understand patterns of community integration among older Chinese adults.

## 2 Methods

### 2.1 Study design

**Figure 2:**
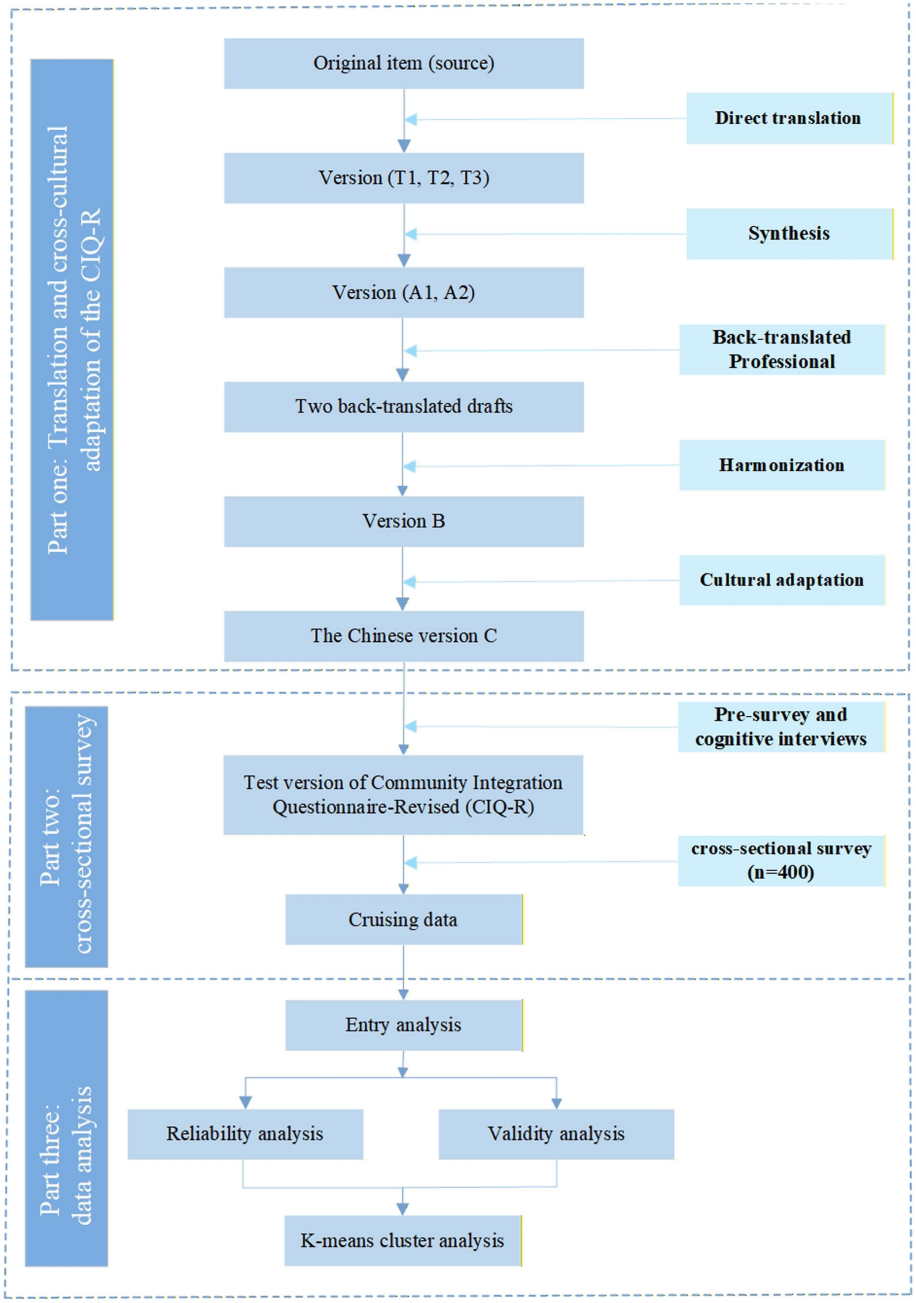
Study design and analytic framework.

A cross-sectional study was conducted in Guizhou Province, China, to translate and culturally adapt the CIQ-R, evaluate its psychometric properties, and characterize patterns of community integration among older adults. All procedures adhered to the principles of the Declaration of Helsinki and were approved by the Medical Ethics Committee of the Affiliated Hospital of the Zunyi Medical University (KLL-2023-228). A detailed flowchart of the study process is presented in Figure 2.

### 2.2 Instruments

#### 2.2.1 Scale version

The CIQ-R includes four subscales: Family Integration, Social Integration (participation in activities and relationships outside the household), Productivity (engagement in employment, education, and volunteer work), and Electronic Social Networking (ESN). The addition of three ESN items strengthens the instrument’s ability to capture technology-mediated participation, a growing component of social integration among older adults. In total, the CIQ-R includes 18 items assessing 16 variables.

Most items are scored from 0 to 2. Productivity-related items range from 0 to 5 depending on employment status, educational participation, and involvement in volunteer activities; these scores are subsequently combined into a single productivity variable. Subscale scores are calculated for Family Integration (0–12), Social Integration (0–10), Productivity (0–7), and ESN (0–6). Summing these yields a composite score ranging from 0 to 35, reflecting the extent of involvement in a wide range of community activities, including financial management, shopping, childcare, cooking, household tasks, community mobility, social interactions, employment, education, volunteer work, and technology-supported communication (23). Higher scores indicate stronger community integration.

#### 2.2.2 Translation

##### 2.2.2.1 Scale translation

The CIQ-R was translated into Chinese with permission from the original developers (23) and in accordance with the AAOS Guidelines for Cross-Cultural Adaptation (29).

(1) Forward translation: Three bilingual native Chinese nurses (two with master’s degrees and CET-6 English proficiency; one clinical nurse specialist with a doctoral degree and extensive international academic experience) independently produced three Chinese versions (T1–T3).

(2) Synthesis: A bilingual nurse synthesized T1–T3 into version A1. Two researchers, together with the translators, reviewed A1 against the original scale and reached consensus on wording, generating version A2.

(3) Back-translation: Two translators (one nursing expert and one master’s student), both blinded to the scale, independently back-translated A2. The research team compared the back-translations with the original and resolved discrepancies, producing the final back-translated version (B).

##### 2.2.2.2 Cultural adaptation

Eight experts with extensive experience or high academic qualifications in sociology or nursing reviewed Chinese version B during a group meeting. We selected experts based on three criteria: research interests in nursing, geriatrics, sociology, or psychology; over 10 years of professional experience; with recent publications related to geriatric management. Each expert independently evaluated the scale using their clinical and professional knowledge, focusing on item accuracy, language clarity, and cultural relevance, and proposed modifications as needed. They also rated each item’s relevance on a four-point scale from “irrelevant” to “strongly relevant.” The researcher synthesized these ratings and suggestions, calculated content validity, and revised the scale accordingly, producing the Chinese version C.

### 2.3 Pre-survey

A preliminary investigation took place in a community in Zunyi City, where we invited a randomly selected group of 30 older adults to participate in cognitive interviews. We set the age criterion at ≥60 years in line with China’s national ageing policy and the official definition of older adulthood (30). Participants also needed to have lived in the community for at least 6 months, communicate in Mandarin, and demonstrate adequate comprehension. We excluded individuals with severe cognitive or psychiatric disorders or those receiving palliative care with a life expectancy under 6 months.

The interview guide explored participants’ overall impressions of the questionnaire, items they found confusing, wording that felt inappropriate, and suggestions for improving unclear or culturally mismatched descriptions. All interviews were recorded in full and transcribed verbatim. Two trained researchers independently reviewed and coded the transcripts, focusing on recurrent issues related to clarity, cultural relevance, and readability. Discrepancies were resolved through discussion, and full consensus was reached.

Recurring feedback was systematically mapped to specific items using a revision matrix to guide item refinement. Based on these insights, the Chinese Version C was revised through wording adjustments, simplification of expressions, and replacement of culturally mismatched examples.

### 2.4 Setting and participants

The study was conducted in five community healthcare centers in a district of Zunyi, China, from April to July 2024. The inclusion and exclusion criteria were identical to those used in the cognitive interviews. Following cross-cultural adaptation guidelines, the sample size was determined to be at least 10 times the number of scale items. The Chinese test version of the CIQ-R contains 18 items (16 variables), and accounting for a 20% attrition rate, the estimated minimum sample size was 216 participants. All participants provided informed consent and voluntarily took part in the study.

### 2.5 Data collection

Data collection was implemented following a standardized, ethically aligned protocol to ensure participant safety, data accuracy, and procedural rigor.

Pre-survey Preparation: One week before fieldwork, the research team coordinated with community public health officers to finalize logistics, verify venue readiness, and ensure consistent understanding of study requirements. All investigators received standardized training to ensure consistency in communication methods and ethical practices. The day before data collection, the community leader informed the participants about the survey schedule.

Participant Recruitment and Informed Consent: On the survey day, trained investigators screened older adults based on the predefined inclusion and exclusion criteria. The study purpose, procedures, potential risks, and benefits were explained in clear, plain language. Written informed consent was obtained from all participants, who were reminded of their right to withdraw at any stage without penalty. Given that some community-dwelling older adults may present mild cognitive limitations, investigators conducted brief comprehension checks to determine capacity for consent, while still encouraging the older adult to complete the questionnaire independently whenever feasible, and only participants who demonstrated sufficient comprehension during screening were included in the final analysis.

Survey Administration: Questionnaires were self-administered and required approximately 6 min to complete. To minimize social desirability influence and protect privacy, all participants responded independently, proxy responses were not permitted, and investigators provided only neutral clarification when necessary. Each questionnaire was labeled with a non-identifiable code, and data were stored securely to maintain confidentiality.

Appreciation: After completing the survey, participants received a small gift (valued at 10 USD) as a token of appreciation for their time and contribution.

### 2.6 Statistical analysis

All analyses were conducted using SPSS 26.0, AMOS 28.0, and R (latest stable version) for clustering procedures. Categorical variables were presented as frequencies and percentages (%), while quantitative data conforming to a normal distribution were expressed as mean ± standard deviation (*x̄* ± *s*). A *p*-value < 0.05 was considered statistically significant.

#### 2.6.1 Correlation analysis

Item analysis was conducted using the critical ratio (CR) and correlation coefficient methods to identify and exclude low-correlation items. For the CR method, participants were ranked by total questionnaire scores. The top 27% of scores were classified as the high group, and the bottom 27% as the low group. A CR > 3.000 was considered indicative of good item discrimination (31). The correlation coefficient method assessed the representativeness of each item by calculating the Pearson correlation coefficient between individual items and the total score. A coefficient ≥ 0.40 was considered acceptable (32). Additionally, if an item has poor relevance and the Cronbach’s alpha coefficient increased after deleting an item, the item was considered inconsistent with the other items and was excluded (33).

#### 2.6.2 Exploratory factor analysis

The EFA was conducted to explore the underlying factor structure and item discrimination of the CIQ-R (34). Sampling adequacy was assessed using the Kaiser–Meyer–Olkin (KMO) test and Bartlett’s test of sphericity, with KMO > 0.80 and *p* < 0.05 indicating suitability for factor analysis (35).

#### 2.6.3 Content validity

Eight experts rated the relevance of each item to community integration on a 4-point Likert scale (1 = not relevant, 4 = highly relevant). Item-level content validity (I-CVI) and average scale-level content validity (S-CVI) indices were calculated. An I-CVI ≥ 0.78 and S-CVI ≥ 0.90 indicated good content validity (36).

#### 2.6.4 Construct validity

Confirmatory factor analysis (CFA) was performed based on EFA results. Model fit was evaluated using *χ*^2^/df < 5, RMSEA < 0.08, and TLI, CFI > 0.90 as acceptable thresholds (34).

#### 2.6.5 Reliability analysis

Internal consistency of the CIQ-R was assessed using Cronbach’s alpha. A threshold of *α* ≥ 0.70 was considered acceptable (37).

#### 2.6.6 *K*-means clustering analysis

*K*-means clustering was performed to identify distinct subgroups of participants based on responses to the culturally adapted CIQ-R. The optimal number of clusters (K) was determined using the Elbow Method. Participants were assigned to the cluster with the nearest centroid, and centroids were iteratively updated until assignments stabilized (38). Analyses were conducted in R (version 4.5).

## 3 Results

### 3.1 Participant characteristics

In this study, a total of 408 participants were approached, and 400 completed the questionnaire, yielding a response rate of 98.04%. The majority of the respondents were female (60.0%) and the mean age of the participants was 74.3 ± 8.2 years (Table 1).

**Table 1:** Participants’ demographic characteristics (*N* = 400).

Characteristics	*N*	%
Gender		
Male	160	40.00
Female	240	60.00
Age		
60–74	187	46.75
75–89	205	51.25
>89	8	2.00
Residence		
City Center	393	98.25
Peri-urban	7	1.75
Village	0	0.00
Marital status		
Married	308	77.00
Divorced	9	2.25
Widowed	81	20.25
Unmarried	2	0.50
Education level		
Illiterate	56	14.00
Primary school	123	30.75
Junior high school	120	30.00
Senior high school	74	18.50
University	27	6.75

### 3.2 Item analysis

The results showed that the correlation coefficients *r* ranged from 0.405 to 0.876 (*p* < 0.001), except for item 2.4(0.008), indicating that item 2.4 showed poor item–total correlation. The results of discriminant and correlation analyses are presented in Table 2.

**Table 2:** Item analysis results.

Item	CR	Corrected item-total correlation
1.1 Who usually does the shopping for groceries or other necessities in your household?	17.180***	0.791***
1.2 Who usually prepares meals in your household?	19.475***	0.816***
1.3 In your home who usually does the normal everyday housework?	17.857***	0.811***
1.4 Who usually cares for the children in your home?	20.263***	0.836***
1.5 Who usually plans social arrangements such as get-togethers with family and friends?	10.390***	0.637***
1.6 Who usually looks after your personal finances, such as banking and paying bills?	9.925***	0.647***
2.1 Approximately how many times a month do you usually participate in shopping outside your home?	8.882***	0.634***
2.2 Approximately how many times a month do you usually participate in leisure activities such as movies, sports, restaurants, etc.?	8.336***	0.551***
2.3 Approximately how many times a month do you usually visit your friends and relatives?	7.583***	0.539***
2.4 When you participate in leisure activities do you usually do this alone or with others?	0.403	0.008
2.5 Do you have a best friend in whom you confide?	6.962***	0.464***
3.1 How often do you travel outside the home?	7.382***	0.478***
3.2 Productivity (work/school/volunteering)	4.648***	0.405***
4.1 How often do you write to people for social contact using the Internet (e.g., email, social networking sites)?	13.514***	0.716***
4.2 How often do you talk to people for social contact using an online video link (e.g., Skype, FaceTime)?	14.187***	0.733***
4.3 How often do you make social contact with people by talking or text messaging using your phone?	9.800***	0.617***

***p < 0.001; CR (critical ratio).

### 3.3 Internal consistency

Cronbach’s alpha for the CIQ-R was 0.816, indicating good internal consistency and acceptable reliability.

### 3.4 Exploratory factor analysis

KMO was 0.808 and Bartlett’s test was significant (*χ*^2^ = 2364.481, *p* < 0.001). The four-factor solution was obtained after varimax rotation (61.25% variance explained). All items showed primary loadings ≥ 0.50 without notable cross-loadings. One item (Item 2.3) loaded on a different factor than in the original scale. Factor loadings are shown in Table 3.

**Table 3:** Factor loading of items.

Item	Home integration	Electronic social networking	Social integration	Productivity
Eigen values	4.687	2.032	1.274	1.197
Percentage of variance	31.249	13.549	8.493	7.959
Factor loadings				
1.1 Who usually does the shopping for groceries or other necessities in your household?	0.779			
1.2 Who usually prepares meals in your household?	0.857			
1.3 In your home who usually does the normal everyday housework?	0.841			
1.4 Who usually cares for the children in your home?	0.893			
1.5 Who usually plans social arrangements such as get-togethers with family and friends?	0.621			
1.6 Who usually looks after your personal finances, such as banking and paying bills?	0.576			
2.1 Approximately how many times a month do you usually participate in shopping outside your home?			0.630	
2.2 Approximately how many times a month do you usually participate in leisure activities such as movies, sports, restaurants, etc.?			0.787	
2.3 Approximately how many times a month do you usually visit your friends and relatives?				0.547
2.5 Do you have a best friend in whom you confide?			0.566	
3.1 How often do you travel outside the home?				0.646
3.2 JOBSCHOOL Variable Scoring: Please check the answer below that best corresponds to your current (during the past month) work situation: In the past month, how often did you engage in volunteer activities?				0.724
4.1 How often do you write to people for social contact using the Internet (e.g., email, social networking sites)?		0.872		
4.2 How often do you talk to people for social contact using an online video link (e.g., Skype, FaceTime)?		0.883		
4.3 How often do you make social contact with people by talking or text messaging using your phone?		0.739		

### 3.5 Content validity

The S-CVI of the CIQ-R was 0.889, indicating that the content validity of the scale was acceptable, and the I-CVI of each item was between 0.875 and 1.000, which indicated that the content validity of each item was satisfactory.

### 3.6 Construct validity

The CFA was used to test the fit of the scale. The results showed a good fit of the scale where $\chi^{2}$/df = 3.194, CFI = 0.920, RMR = 0.048, TLI = 0.900, RMSEA = 0.074.

### 3.7 Cluster analysis

**Figure 3:**
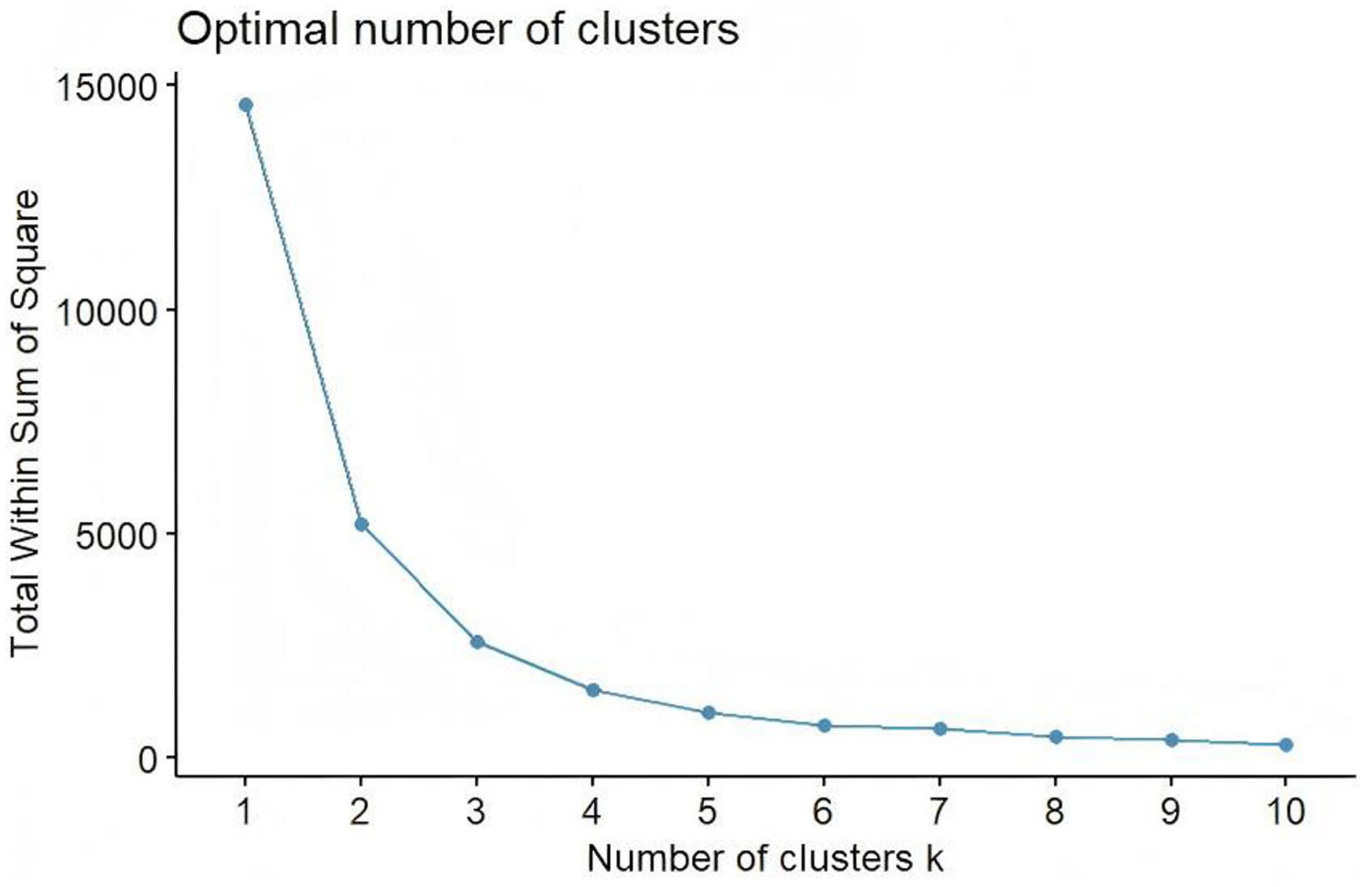
Number of clusters *k*.

**Figure 4:**
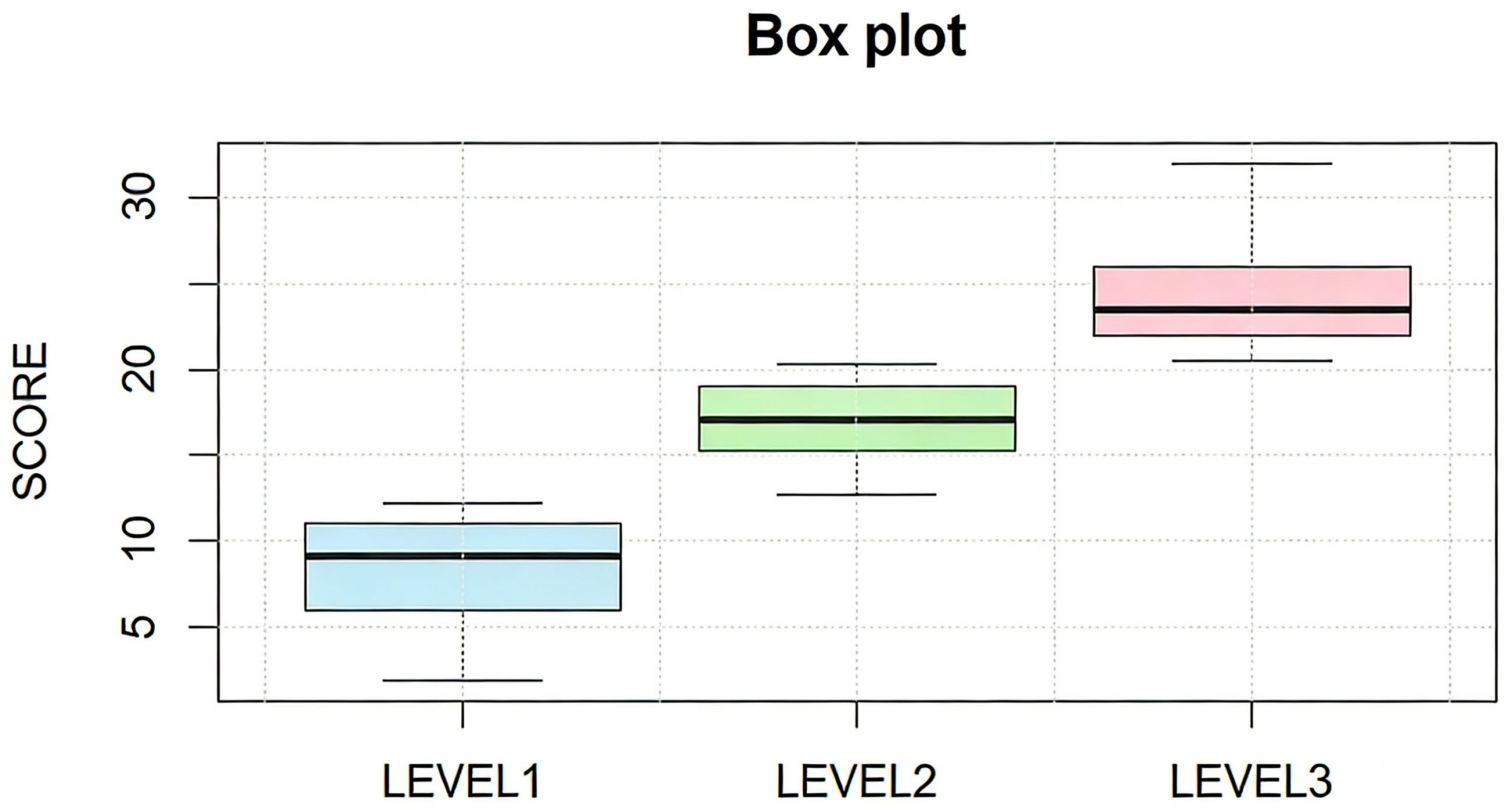
Cluster analysis box diagram.

The Elbow Method supported a three-cluster solution (Figure 3). *K*-means classified participants into Low (level 1), Moderate (level 2), and High community integration (level 3) groups (Table 4). The Kruskal–Wallis test confirmed overall differences (*H* = 339.188, *p* < 0.001), with all pairwise comparisons remaining significant (*p* < 0.001). The empirical cut-points values for community integration were determined as follows: 13.25 for Low to Moderate, and 21.00 for Moderate to High. Box plots showed clear separation across clusters (Figure 4).

**Table 4:** Cluster analysis results (*N* = 400).

Level	*N*	Mean ± SD	Minimum	Maximum	Centers	*H*	*P*
1 (Low)	68	9.063 ± 3.24	2	13.25	9.06	339.188	<0.001
2 (Moderate)	179	17.647 ± 2.152	13.5	21	17.65		
3 (High)	153	24.677 ± 2.662	21.25	32	24.68		

**Figure 5:**
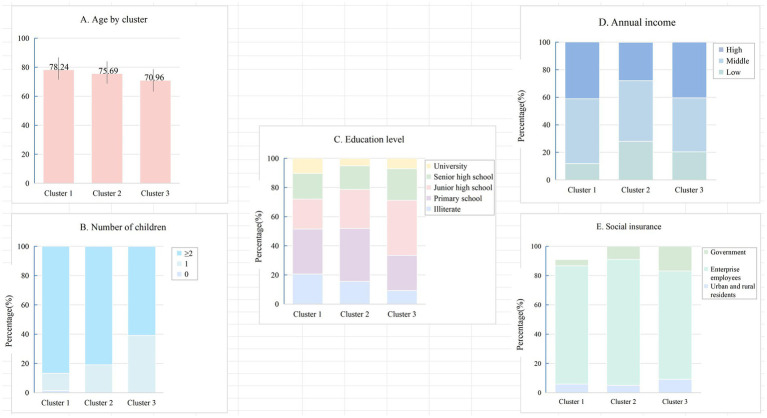
Cluster-specific characteristics. **(A)** Age; **(B)** Number of children; **(C)** Education level; **(D)** Annual income; **(E)** Social insurance type.

Five demographic variables differed significantly across clusters (Table 5). Age decreased progressively across groups (*F* = 25.98, *p* < 0.001), with Cluster 1 being oldest and Cluster 3 youngest. Education level varied (*χ*^2^ = 18.186, *p* = 0.020), with higher education more common in Cluster 3. Number of children also differed (*χ*^2^ = 30.442, *p* < 0.001), with fewer participants reporting ≥2 children in Cluster 3. Annual income varied across clusters (*χ*^2^ = 11.682, *p* = 0.020), with Cluster 3 showing higher representation in high income level. These patterns are illustrated in Figures 5A–E. Other demographic variables showed no significant differences and are not presented.

**Table 5:** Characteristics of participants by cluster group (*N* = 400).

Variable	Cluster 1 (*n* = 68)	Cluster 2 (*n* = 179)	Cluster 3 (*n* = 153)	*χ*^2^/*F*(df)	*p*
Age (mean ± SD)	78.24 ± 8.60	75.69 ± 6.93	70.96 ± 8.19	*F*(2,397) = 25.98	<0.001
Education level *n* (%)				18.186	0.020
Illiterate	14 (20.6%)	28 (15.6%)	14 (9.2%)		
Primary school	21 (30.9%)	65 (36.3%)	37 (24.2%)		
Junior high school	14 (20.6%)	48 (26.8%)	58 (37.9%)		
Senior high school	12 (17.6%)	29 (16.2%)	33 (21.6%)		
University	7 (10.3%)	9 (5.0%)	11 (7.2%)		
Number of children *n* (%)				30.442	<0.001
0	1 (1.5%)	0 (0.0%)	0 (0.0%)		
1	8 (11.8%)	34 (19%)	60 (39.2%)		
≥2	59 (86.8%)	145 (81.0%)	93 (60.8%)		
Social insurance *n* (%)				12.305	0.015
Urban and rural residents	4 (5.9%)	9 (5.1%)	14 (9.2%)		
Enterprise employees	61 (89.7%)	153 (85.4%)	113 (73.9%)		
Government	3 (4.4%)	17 (9.5%)	26 (17.0%)		
Annual income *n* (%)				11.682	0.020
Low	8(11.8%)	50(27.9%)	31(20.3%)		
Middle	32(47.1%)	79(44.1%)	60(39.2%)		
High	28(41.2%)	50(27.9%)	62(40.5%)		

## 4 Discussion

This study translated and culturally adapted the CIQ-R for Chinese older adults, confirming that the revised instrument demonstrates solid reliability, content validity, and a stable factor structure. Compared with existing CIQ-R studies—most of which were conducted in clinical or rehabilitation settings in Malaysia (27) and Italy (28), with other studies reporting issues such as cross-loadings (39) and low item loadings (40)—the revised Chinese version performed well among community-dwelling older adults. This suggests that the scale aligns more naturally with this population.

During adaptation, we replaced digital platforms unfamiliar to older adults with WeChat and Sina Weibo, which improved clarity without altering the underlying construct. Most items behaved as expected, though item 2.4 (“Leisure activities alone/others?”) stood out—the item did not discriminate well, and the scale became more coherent after its removal. Similar problems have surfaced in other cultural contexts, which suggests that distinguishing solitary from shared leisure activities may not translate cleanly across age groups or cultural contexts. The final internal consistency aligned closely with that of the Malay version and surpassed the Italian validation.

The factor structure held up largely as anticipated, aside from one interesting shift. Item 2.3 (“visiting family/friends”) aligned with Productivity rather than Social Integration. Such shifts are common in cross-cultural validation when behaviors serve multiple cultural functions (41). This pattern is consistent with common features of Chinese family life. A visit to a relative often involves caretaking, errands, or keeping intergenerational obligations—not merely social pleasure (42). Such differences remind us that cultural adaptation works best when we privilege conceptual meaning over literal translation. The CFA results confirmed that keeping this item within the Productivity domain produces a coherent and defensible model.

We then used a data-driven approach to classify levels of community integration (43). Instead of relying on arbitrary cutoffs, the clustering analysis revealed three distinct patterns anchored at CIQ-R scores of 13.25 and 21. These patterns echo gradients commonly observed in functional ability, social participation, and mental health (44, 45). These empirical cut-points provide a pragmatic basis for stratifying community-dwelling older adults according to integration risk in public health screening and community service planning. Older adults falling into the low-integration group tended to be older, less educated, and less financially secure; these trajectories mirror the logic of the Cumulative Advantage framework (46), where early advantages accumulate and widen over time. Qualitative studies from China paint a similar picture: many older adults experiencing financial pressure or limited education describe shrinking networks, fading confidence in public spaces, and the feeling of “slipping out of the circle” as communities modernize faster than individuals can adapt (47). Integrating insights from existing qualitative studies allows us to situate the observed patterns, particularly the vulnerabilities of the low-integration group, in the lived experiences of older adults navigating rapidly shifting social environments. Beyond classification, our findings also highlight the mechanism through which community integration supports healthy ageing. Within the WHO framework, functional ability emerges when intrinsic capacity is reinforced by an enabling environment. This study found that older adults with higher integration displayed richer social ties, more consistent engagement, and broader activity involvement—they are likely to be associated with better mobility, sharper cognition, and steadier emotional states. Fieldwork and qualitative interviews in Chinese neighborhoods have shown similar dynamics: whether through social activities, family support, enhanced community environments, or digital communication, such engagement can soften loneliness and help preserve a sense of identity (48). Seen together, our findings position community integration as more than a social indicator; it becomes a pathway through which environments shape trajectories of ageing.

By contextualizing the CIQ-R for use in China, we reduce cultural bias and offer a practical tool for population-level assessment, program evaluation, and targeted interventions. Policymakers can use score thresholds to identify older adults who may benefit from more frequent social opportunities, educational activities, psychological support, or digital literacy programs. Tailored strategies informed by cluster profiles offer a clearer way to match services with need and to strengthen ageing-related public health efforts.

This study has limitations. The sample was drawn from a single province, which may constrain generalizability. Reliance on self-report measures may introduce recall or social desirability bias. Although all participants completed the questionnaire independently and anonymously, subtle response distortions cannot be fully ruled out. Mild cognitive limitations may also influence how certain items are interpreted, even when comprehension checks appear satisfactory. Future studies integrating objective functional measures, longitudinal and mixed-methods designs will further strengthen causal inference and public health applicability.

## 5 Conclusion

This study advances the cross-cultural assessment of community integration by refining the factor structure of the CIQ-R and identifying meaningful integration profiles among Chinese older adults. From a public health perspective, the adapted CIQ-R enhances measurement precision, captures culturally shaped interpretations of social engagement, and provides actionable tools for population-level monitoring of social functioning and the design, evaluation, and targeting of community-based ageing interventions in rapidly ageing societies.

## Data Availability

The raw data supporting the conclusions of this article will be made available by the authors, without undue reservation.
